# A settler physician perspective on Indigenous health, truth, and reconciliation

**Published:** 2018-07-27

**Authors:** Denise Jaworsky

**Affiliations:** 1 Northern Medical Program, University of Northern British Columbia, British Columbia, Canada; 2 Department of Medicine, University of British Columbia, British Columbia, Canada

## Abstract

This brief report presents one settler physician’s perspectives on our responsibility to engage in reconciliation and decolonize our healthcare institutions. It draws from existing literature to identify key actions for reconciliation in health care. These include i) engaging Indigenous peoples as leaders and equal partners in developing health interventions, ii) increasing our awareness and education around the colonial history and settler presence in Canada, including our role in the ongoing oppression of Indigenous peoples, iii) providing services in ways that recognize and mitigate colonial determinants of health, and iii) practicing cultural safety at an individual level and advocating for it at a structural level. These actions can be realized through educational interventions and ongoing reflexivity among medical trainees and practicing physicians.

My family has been in Canada for four generations and I embrace my Canadian identity. Canada is my home, but not my traditional territory. When my family left Japan, they settled in Canada, and so we are settlers. I am a general internist in Northwestern British Columbia and I provide services to a mix of non-Indigenous individuals and Indigenous peoples from multiple nations. I predominantly work in a hospital and clinic setting in the city of Terrace which is located on Tsm’syen territory.

As a settler physician in a settler colonial state, I have learned about related social determinants of health. In medical school we were taught to apply this lens when considering the health of Indigenous peoples, but where our education has failed us is in understanding our role in the ongoing colonialization and racialization of Indigenous peoples in Canada. As a Canadian physician, this is something that I must come to terms with. Regardless of where we practice, physicians in Canada will provide care to Indigenous peoples whose health and wellness have been inordinately impacted by settler colonialism.

The Truth and Reconciliation Commission of Canada describes reconciliation as “coming to terms with events of the past in a manner that overcomes conflict and establishes a respectful and healthy relationship among people, going forward.”^1^ As Canadians, we have an obligation to embrace our responsibilities in reconciliation. As physicians, in particular, we must first understand how the care we provide may be unsafe and begin our own paths to reconciliation and cultural safety in healthcare.

## Towards truth

While we may be familiar with the determinants of health and can easily recognize, for example, how poverty and housing insecurity can predispose communities to poor health outcomes, we may not, amidst our busy clinical duties, reflect on upstream political and historical factors driving these determinants. Settler colonialism has been implicated in contributing to poor health among Indigenous peoples in numerous ways since the beginning of contact. These include the introduction of foreign infectious diseases, drugs and alcohol, interference with traditional hunting and gathering leading to dependence on settler food, contamination of natural resources by heavy metals and industrial waste, and lands dispossession.^2^ Ojibwe researcher, Earl Nowgesic describes the Indian Act, which has been implicated in the state-legislated control and erasure of Indigenous identity, as a structural factor affecting the health of First Nations peoples.^3^ By conceptualizing settler colonialism as a distal determinant of health we can understand how it creates environments and policies that contribute to poor health at a structural level.^4-6^ It is this colonialism entrenched within our political, economic and legal systems that enables the ongoing land dispossession, cultural assimilation and socioeconomic discrepancies which negatively affect the health of Indigenous peoples in Canada.

In addition to the ongoing effects of settler colonialism on the health of Indigenous peoples in Canada, their experiences within the dominant healthcare system have been variable and experiences of racism continue to act as a barrier to seeking care.^6^ This may be unintentional and many healthcare professionals may be oblivious to how our interactions with Indigenous patients may leave them feeling devalued. But intention does not alter the outcome that Indigenous peoples often receive substandard or unequal care based on their race. An overt example of this is the case of Brian Sinclair who died after 34 hours in a Winnipeg emergency department waiting room without receiving any medical care.^7^ An example where healthcare providers may be oblivious to the impact of racism is illustrated by Browne et al. who describe how First Nations women felt the need to shift their appearance and behaviours into those that they felt were more credible and worthy of receiving medical care.^8^

As physicians, we are accustomed to helping people. We have the privilege of providing care to people in their most vulnerable moments. However, we can become so immersed in our role as healer that we fail to recognize that we may be supporting the ongoing colonization of Indigenous peoples through paternalistic models of care. This paternalism is driven by racist assumptions that Indigenous peoples must be “civilized” or “brought up” to the standard set by the settler state. This paternalism manifests frequently in encounters between Indigenous patients and settler physicians. It manifests when we do not ask about traditional medicine; when we do not understand that an individual’s medical appointment may not be a priority because their family or community needs them; when we extract pregnant women from their communities on the assumption that hospital-based care is more important than birthing in one’s traditional territory; and when we fail to recognize Indigenous beliefs about healing and wellness as both relevant and legitimate. We must ask ourselves whether this paternalism and cultural superiority within medicine differs from the ideology that allowed our nation to assert colonial control over Indigenous peoples through residential schooling, the “sixties scoop,” and current child welfare structures under the pretense of ‘protecting’ Indigenous children.

As physicians who provide care to Indigenous peoples, it is not enough to understand that these populations may be at risk for poor health outcomes. We must educate ourselves about Canada’s colonial history, understand how settler colonialism is a determinant of health and reflect on how our institutions and practices may be perpetuating inequity in health. By confronting these truths, reconciliation begins to take meaning.

## Towards reconciliation

Reconciliation can have very different meanings to different people and some of these views are conflicting.^1^ As settler physicians, we need to envision a path towards reconciliation that is shaped by our experiences and the relationships we build with our Indigenous patients. Through my own reflections, guided by the mentorship of Indigenous physicians, Elders, scholars and community advocates, I offer several suggestions for other settler physicians. These are not meant to be comprehensive or to replace the critical dialogue lead by Indigenous physicians, but rather to stimulate discussion and add a settler perspective in support of their work.

### Honour Indigenous expertise

As settler physicians or policy makers, we must acknowledge that no degree or fellowship can make us experts in the health of communities to which we do not belong. Instead, we must humbly learn from our colleagues who are Indigenous physicians, Elders, knowledge keepers, healthcare providers, caregivers, scholars, and community members. We must allow our Indigenous colleagues to help us decolonize how we deliver healthcare and recognize that it is not the “Indian Problem” that needs fixing. We must modify our existing medical education system to better attract and support Indigenous medical students and residents so they can thrive as physicians in a learning environment that honours rather than threatens their Indigenous identity and is free of racism and discrimination. We must also recognize and create space for Indigenous leadership in health policy and program development rather than preserving norms of colonial control.

### Evaluate our role in colonialism

As a Canadian physician, I feel fortunate to belong to one of the most privileged professions, frequently benefitting from the associated credibility, respect, income security, and social status. Yet our curricula often fail to create a space for anti-racism, anti-oppression, and anti-colonialism training or for the discussion and recognition of our own privilege.^9^ In the field of teacher education, Haudenausanee scholar Martin Cannon identifies “privileged learners,” and this concept can be extended to medical education where transformative change can only occur once we recognize ourselves as beneficiaries of colonialism, accept “collective responsibility for our complicity in social inequality,” and understand how our social advantage can be unwittingly derived from the oppression of others.^10^

### Understand colonialism as a determinant of health

Indigenous scholars such as Earl Nowgesic and Charlotte Reading demonstrate the importance of contextualizing healthcare through colonial determinants of health as a step towards self-determination in healthcare.^3,11^ A discussion of determinants of health is mandated within Canadian medical school curricula, however the impact of settler colonialism rarely finds a place in this already limited discourse. This discussion is essential in physician training and needs to include a frank description of the historical realities of settler colonialism in Canada. This will enable us to become physicians who can critically reflect on our own privilege and use our collective voice to dismantle institutional racism and advocate for structural changes that will address underlying colonial determinants of health. In addition to treating disease, we must play a role in mitigating the factors that contribute to ill health in the first place.

### Embrace cultural safety and advocate for it at an institutional level

In 2013, the Royal College of Physicians and Surgeons of Canada (RCPSC) formally identified the need for physicians to embrace cultural safety and engage in a process of self-reflection so we can modify our own practices.^12^ In 2017, based on the recommendation of the Indigenous Health Advisory Committee, the RCPSC subsequently mandated that Indigenous health become a part of all postgraduate medical education.^13^ The concept of cultural safety was first described by Maori scholars, but many Indigenous physicians in Canada have been at the forefront of bringing this into Canadian medical education.^14-16^ Rather than focusing on differences among cultures, a cultural safety model calls for critical reflection among service providers to identify power imbalance that can exist among groups at a systemic level and to subsequently begin to dismantle these hierarchies. A cultural safety approach in healthcare can lead to the recognition of how policies and practices often create inequities and marginalize populations.^17^ By advocating for the institutionalization of cultural safety training and practices, we can begin to transform our working environments into places where fundamental human rights of Indigenous people are respected and they receive the best possible healthcare in a manner that is free of racism.^18^

## Conclusion

As physicians, we may perpetuate health inequities through ignorance about colonial determinants of health, ignoring our role in perpetuating power imbalances, complicity in institutional racism, and failing to question paternalistic models of care. To move beyond this, we need to i) engage Indigenous peoples as leaders and equal partners in developing health interventions, ii) increase our awareness and education around the colonial history and settler presence in Canada, including our role in the ongoing oppression of Indigenous peoples, iii) provide services in ways that recognize and mitigate colonial determinants of health, and iv) practice cultural safety at an individual level and advocate for it at a structural level. In doing so we may finally find ourselves on a path towards reconciliation in healthcare.

## Appendix A Glossary of terms

Settler colonialism: a form of colonialism where Indigenous populations are intentionally displaced and replaced by a foreign settler society that ultimately develops its own identity and sovereignty.^19^

Distal determinant of health: also known as ‘upstream’ determinants of health, distal determinants of health are the social, political, economic and cultural factors that impact people’s lifetime exposure to proximal (physical, behavioural, psychosocial and biological) determinants of health.^20^

Indian Act: an act of Canadian parliament that was first passed in 1876 that was designed to facilitate service and program delivery for First Nations peoples and assimilate them into the settler society. The Indian Act controls who is considered to have Indian ‘status’ and the associated entitlements from the Canadian government.^21^
